# Proactive Innovation in a Prolonged Conflict Setting: Facing COVID-19 in a Specialized Cancer Hospital in Palestine

**DOI:** 10.3389/fpubh.2022.873219

**Published:** 2022-04-01

**Authors:** Ali Sabateen, Merette Khalil, Munia Abu El Hawa, Richard Peeperkorn, Awad Mataria, Hamid Ravaghi

**Affiliations:** ^1^Head of Infectious Diseases, Augusta Victoria Hospital, Jerusalem, Palestine; ^2^Universal Health Coverage and Health Systems Department, World Health Organization Regional Office for the Eastern Mediterranean, Cairo, Egypt; ^3^World Health Organization, Jerusalem, Palestine

**Keywords:** COVID-19, cancer, innovation, infectious disease, hospital resilience, emergency preparedness and response

## Abstract

The prolonged ongoing conflict in Palestine exacerbated socioeconomic conditions and weakened the health system, complicating the management of COVID-19 pandemic, especially for cancer patients who are doubly-at risk. Augusta Victoria Hospital (AVH) is Palestine's only specialized cancer hospital, receiving patients from the Gaza Strip and the West Bank for oncology, nephrology, hematology, and radiotherapy. AVH's preparedness measures enabled its agile response. These proactive and innovative preparedness measures included: implementing a facility-level preparedness and response plan; utilizing multidisciplinary team-based and evidence-informed approaches to decision making; prioritizing health workers' safety and education; establishing in-house PCR testing to scale up timely screenings; and accommodating health workers, patients, and their relatives at hospital hotels, to maintain daily, continuous and critical health care for cancer patients and limit the spread of infection. At the facility-level, the biggest challenge faced by AVH was continuing essential and daily care for immunocompromised patients while protecting them from potential infection from relatives, hospital staff and other suspected patients. At the national level, the lack of preparedness, inequalities in vaccine distribution, political instability, violence, delays in obtaining medical exit permits to reach Jerusalem, weakened AVH's response. AVH's flexible financing, hospital accreditation, and strong leadership and coordination enabled its agility and resilience. Despite compiling challenges, the hospital's proactive and innovative interventions minimized the risk of infection among two high-risk groups: the immunocompromised patients and their health workers, providing invaluable lessons for health facilities in other fragile-and-conflict-affected settings.

## Introduction

The management of the COVID-19 pandemic in Palestine is challenged by the prolonged and ongoing conflict, exacerbating fragility of socio-economic and health systems (1). For the last 14 years, the Gaza strip has been under siege, while restricted mobility and limited resources obstruct Palestinian's access to health care in the West Bank including in East Jerusalem (2, 3) (Box 1).

Box 1 **A snapshot of Palestine's protracted crisis.**

According to the latest Humanitarian Response Plan (HRP-oPT 2022), approximately 2.1 million Palestinians remain vulnerable due to the ongoing protracted crisis (4). “The West Bank is divided into Areas A, B and C, in addition to the Israeli-controlled areas of H2 in Hebron and East Jerusalem, presenting further difficulties for the cohesiveness of the health system and for access for staff, ambulances, patients and relatives (5).”

In 2021, Palestine witnessed the most serious escalation of hostilities with extensive clashes in Gaza and East Jerusalem since 2014 (4). UN reports state that Israeli settlement activity and related violence, loss of land, destruction of property and movement restrictions, threats of eviction, and restricted access to basic services and livelihoods contribute to a coercive environment which deny Palestinians their basic rights and pressure some to leave their homes (4, 5). Further to this, the conflict caused significant damage and loss of life, increased aid dependency and exacerbated poverty (5). The economy remained almost stagnant in the first half of 2021, with unemployment reaching 44.7 per cent and poverty almost 60 per cent (4).

Palestine is considered as a complex and challenging environment to provide health services due to the political and economic restrictions and territorial barriers (e.g. the presence of checkpoints, West Bank Barrier, settlements, road blocks and the closure of Gaza since 2007) which divide the country. This is further exacerbated by the fiscal financial crisis and the impacts of COVID-19 pandemic.

In July 2021, Palestine recorded one of the highest COVID-19 outbreak rates globally compared to its population size (6). Only three hospitals were designated for COVID-19 nationally, namely: Augusta Victoria, Al-Makassed Islamic Charity, and St. Joseph Hospitals.

The Palestinian Authority refers patients for specialized health care to non-Ministry of Health facilities including Palestinian hospitals in East Jerusalem and the West Bank (Box 1). Located in East Jerusalem with 150 beds and an outpatient chemotherapy unit, Augusta Victoria Hospital (AVH) is Palestine's only specialized cancer hospital, receiving patients from the Gaza Strip and the West Bank for oncology, nephrology, hematology, and radiotherapy. These patients depend on referral to AVH in East Jerusalem for access to full investigation and treatment services, with essential technologies for treatment and investigation of cancer, including facilities for radiotherapy and nuclear medicine scanning as well as some chemotherapy drugs, completely lacking in the rest of the West Bank and Gaza. Prior to the pandemic, AVH provided chemotherapy treatment to 70 to 90 patients per day. Cancer patients are twice as likely to contract COVID-19, with higher rates of complications and hospital readmissions with delayed diagnoses and interrupted treatments (7, 8).

This paper outlines the proactive and innovative interventions implemented by AVH in minimizing the risk and spread of COVID-19 among health workers and immunocompromised patients, along with invaluable lessons learned for health facilities in fragile and conflict-affected settings. This article is extracted from larger regional research on hospitals experiences combatting COVID-19 in the Eastern Mediterranean Region conducted in 2020 which utilized a mixed-methodology triangulating findings from literature review, online survey and 46 key informant interviews. Two key informant interviews from Palestine with AVH's head of infectious diseases unit and a World Health Organization National Technical Officer were extracted for this article. Ethical approval for this type of study is not required by the journal. Ethical approval for this study has been obtained from WHO Regional Ethical Review Committee. These KIIs were conducted in July 2020 and were followed-up four times between June-October 2021 for further updating and validation. This study is informed by a review of gray literature and peer-reviewed publications on hospitals' COVID-19 response in Palestine between Dec 2019–Aug 2021 and in-depth key informant (KI) interviews.

## Hospital Interventions Responding to COVID-19

### Facility-Level COVID-19 Preparedness and Response Plan

Informed by regional and global recommendations, AVH was the first hospital in the country to establish a facility-level COVID-19 preparedness plan, even before the Ministry of Health's lockdowns and COVID-19 national strategies (9). AVH's preparedness strategy was three-pronged: Firstly, hospital workforce training and safety; secondly, timely procurement of supplies and equipment; and thirdly, improving processes and communication.

Firstly, to protect health workers, all AVH staff (including non-clinical employees) were trained on screening and infection prevention and control (IPC) protocols. To reduce the risk of nosocomial infections, AVH implemented two-team rotations, assigning staff into two groups, each continuously monitoring specific departments over 2 weeks before taking a 2-week (off-site) break. In addition, since the first days of the pandemic, AVH distributed surgical and N-95 masks to all hospital workers daily and mandated trainings on the use of personal protective equipment (PPEs).

Secondly, ahead of the first COVID case in Palestine, AVH's proactive approach allowed for adequate procurement of supplies despite: global shortages, sky-rocketing prices and challenges posed by the political and health systems fragility in the country (10). Due to the complexity of the political situation and the difficulties of import/export to Jerusalem and West Bank, there is not a reliable channel to provide surplus PPEs by other countries in the Region. To ensure availability of PPEs, Palestinian hospitals needed to either procured PPEs or await in-kind donations from donors based on needs-assessments. To ensure safe and continuous COVID-19 and cancer care, AVH utilized in-house storage, stocking up as many PPEs, including gloves, gowns, and masks. This surplus safeguarded AVH when other hospitals and health workers in Palestine were facing heightened risks due to shortages and delays in the supply chains due to political unrest and global disruptions to travel and trade.

Thirdly, regarding hospital's processes and communication, AVH's committee communicated a simple and clear triage strategy for suspected and confirmed cases, which could be easily understood and accessible by all hospital staff. During the early months of the pandemic, the daily briefings congregating the senior management team and different types of staff and specialists facilitated: timely decision-making, strategizing, teamwork, collaboration, and accountability. Senior management met daily for briefings and communicated updates with the staff in a timely, clear, and inclusive manner.

### Establishing a Multidisciplinary COVID-19 Committee

AVH established a coronavirus response committee, which was activated when the first case was diagnosed in Bethlehem by mid-April 2020. This committee comprised of Chief Executive and Operations Officers (CEO, COO), and the Medical Director, the infectious disease specialists, members of the infection control committee, pharmacy/supply services, front-liners, and support services like cleaners. To plan the necessary prevention and response strategies, the committee met daily to assess the national, regional, and global situation, including surveillance of neighboring countries, and incidences on the Israeli and Palestinian sides.

### Effective Communication

Internal and external communication ensured AVH is updated and well-informed and strengthened hospital readiness and response to COVID-19. In translating IPC strategies into action, all staff were trained on and understood the process of dealing with suspected cases, including taking samples, transporting swabs, using PPE, disposing of medical waste, isolating patients in the suspected area, donning and doffing processes, and referral pathway for suspected cases, including alerting codes to the IPC committee, hospital CEO, and Ministry of Health. Further, AVH staff utilized WhatsApp groups for rapid and efficient transfer of relevant information and updates to all staff.

Regarding external and risk communication with the public, AVH distributed printed brochures to all patients and their families and referred them to publications and resources by the Ministry of Health on proper prevention and protection practices. Additionally, AVH used videos for risk communication, and shared informational and educational resources on social media (e.g. Facebook) to teach the community about COVID-19 and how to protect themselves from the virus. KIs confirmed that even the ministry of health (MOH) relied heavily on social media, especially Facebook, and frequently updated their websites due to accessible, quick, and widespread reach to the community.

### Incentives for Staff

Despite the challenges of human resources shortage and harsh working conditions in responding to COVID-19, AVH offered health workers additional incentives, including “hardship” allowances, accommodations, and meals. AVH paid to house staff in near-by hotels, and covered expenses for their services, food, and clothes for the first 3 months of the response. As the only cancer hospital in Palestine, and a referral hospital to patients from the West Bank and Gaza, AVH has quite extensive experience in providing accommodation to cancer patients traveling from these territories, due to the frequent closures imposed which restrict access of patients to Jerusalem. The provision of accommodation is one of AVH's most strategic initiatives, prioritized in its planning, budgeting and resource mobilization efforts, in order to ensure continuity of care for immunocompromised patients despite the political restrictions.

### Scaling Up Screening, Testing, Identification, and Diagnosis

AVH used a phone-triage mechanism to screen all incoming and all referred patients prior to their arrival since the hospital does not have an emergency department. Additionally, within the first month of the pandemic, AVH built an in-house laboratory to make PCR available at the hospital. This laboratory was among the first and only laboratory in Palestinian hospitals, despite other governmental and central labs (6). Further to this, AVH designated a COVID “Center” allocating two external areas for suspected and confirmed cases outside the main hospital building, which enabled the continuity of care for critical patients, receiving radiotherapy, chemotherapy, and other services, without the fear of cross-infection or hospital transmission of COVID-19.

### Increasing Surge Capacity and Maintaining Essential Services

To limit infections and maintain essential services, AVH reduced its hospital load by stopping all outpatient clinics, suspending elective surgeries, and postponing non-urgent radiotherapy for a few months. Another strategy in managing surge capacity was shifting either entire teams or departments to support critical and essential services. For example, the department of surgery was shifted to support critical cases; while the empty space (about 12 rooms each with two beds) was used to house staff who spent weeks at a time in the hospital. Further, AVH reassigned the staff of an entire department to a sister hospital in Bethlehem to support cancer patients. The chemotherapy was prepared at AVH and sent to the team in Bethlehem who provided care and worked over 10 hour shifts daily to provide critical care for patients who could not reach the hospital in East Jerusalem. AVH also designated coordinators for chemotherapy, radiotherapy, and nephrology patients, who were responsible to screen patients, who are either referred or scheduled to be admitted through phone-triage daily. The mobile clinic department was reassigned to support with the tele-screening and referrals coordination.

## Enablers to Preparedness

### Financial Flexibility

Prior to the pandemic, AVH was championing the cause regarding antimicrobial stewardship and infection control and was responsible to lead 22 hospitals in minimizing overuse of antibiotics nationally. As the guarantors of a national anti-microbial resistance (AMR) champions fund, AVH shifted this money justifying the interconnectedness of multidrug resistance and infectious disease (including COVID-19) outbreaks. This was beneficial as AVH did not receive additional funding from other sources. This flexible financing further enabled AVH's proactive preparedness including early procurement of supplies and PPEs and accommodation of patients and staff traveling to Jerusalem for medical services.

### Hospital Accreditation

AVH has been accredited by the JCI three times. One of the major strengths of AVH's response was its steadfast implementation of infection control needs assessments and interventions, as part of hospital accreditation strategies. AVH's infection control committee enabled adequate preparedness for COVID-19 outbreak. Moreso, AVH evaluated and learned from previous experiences in managing hospital-acquired outbreaks (e.g., a TB outbreak 2 years prior) which allowed a revamping and strengthening of the facility's IPC mechanisms.

### Strong Leadership and Coordination

The role of senior management was an enabling factor in minimizing the impacts of COVID-19 outbreak at AVH. Hospital senior management utilized a horizontal, supportive, decisive, evidence-based and participatory leadership approach. Trust from the senior management team allowed for quick implementation of preparedness strategies, including creating the COVID-19 center, building the PCR laboratory and training all staff on IPC and triage. For instance, AVH prepared the COVID-19 Center within 1 week, including recruiting and reassigning specialized staff and procuring adequate supplies and ventilators. AVH also ensured that specific entrances and exits were designated for suspected cases. Moreover, the strong leadership capacities enabled the hospital to establish a micro-laboratory to identify cases and monitor the outbreak within 2 weeks. Nowadays, AVH is establishing a vaccination program to increase immunization for high-risk patients, staff, and their relatives.

The senior leadership at AVH, including the CEOs, COO, medical director, led by example and were constantly on the ground, in the early phases, in efforts to prevent the outbreak and protect patients. This was motivational to other hospital health workers. Moreover, AVH's senior management team includes one of the only infectious disease specialists in the country, serving as the director of the hospital's IPC committee.

## Challenges

### Facility Level: Continuous Care for Immunocompromised Patients

Among the top challenges faced by AVH was the type of patients treated. Almost all patients receiving care at AVH are immunocompromised patients, whether receiving oncology or nephrology health services. Generally, patients seeking radiotherapy and oncology treatment at AVH require daily, continuous, and regular health services. A KI highlighted: “*Every single radiotherapy session and every cycle of chemotherapy session is necessary for their survival. All these things were kept in mind when trying not to disrupt essential services.”* Providing these essential health services to patients suspected or infected with COVID-19 presented additional challenges. Starting the early months of the response, additional screenings were conducted daily to prevent transmission of the virus into the hospital. Nevertheless, the hospital had no control over the incoming patients from other hospitals mainly from the Gaza Strip, where it is difficult to perform routine PCR testing (1, 11).

### Health System Level: Lack of National Preparedness Plans and Limited Hospitals' Agility

Contrary to AVH's proactive and innovative approach, KIs confirmed that many hospitals did not have facility-level outbreak preparedness and response plans at the beginning of the pandemic; while at the national level, strategies for hospitals readiness remained preliminary (1, 2, 12, 13). KIs urged hospitals to proactively develop COVID-19, infectious disease outbreak, and all-hazards preparedness and response strategies, in collaboration with the national epidemiological committee and the Ministry of Health.

During the first wave of the pandemic, many hospitals resisted and refused to treat suspected or confirmed COVID-19 patients, resulting in fragmentation in the referral pathways and disruptions to the provision of health services (12). Studies confirmed the limited capacity for isolation and treatment of critical cases in Palestine, with one citing only one isolation hospital designated in the Gaza Strip, AVH and 2 others in Jerusalem and 13 in the remaining part of the West Bank (2). Nowadays, facility and national efforts are still needed to ensure the availability of specialists, critical care beds, testing supplies, PPEs, and vaccines (1, 2, 10). KIs noted that maintaining correct IPC protocols at all times was another challenge as this requires continuous training, commitment, cooperation, devoted efforts and accountability.

Nowadays, due to the global distribution inequalities and difficulties in accessing second-doses of COVID-19 vaccines, one of the major challenges faced by AVH is the difficulties in vaccinating all patients, their companions, and clinical and non-clinical staff members and their relatives (6).

### National Level: Political Instability and Restrictions of the Occupation

Restrictions of movement exacerbated during the global pandemic, resulting in patients and health workers not being able to move smoothly from city to city, which not only inhibited health services accessibility and utilization but also resulted in pressures on staff (10, 11, 14, 15). One study found that prior to the pandemic, only 70% of Palestinians applying for a medical exit permit to access Jerusalem received it in time for their appointments, while 22% were delayed or received no response, and 9% of requests were denied – further exacerbating access to health services (15). As for patients who required daily chemotherapy, radiotherapy, and dialysis, AVH booked hotels in Jerusalem to facilitate access to the hospital. In the first months, KIs recalled some hospital staff slept at AVH for 20 days to manage the outbreak while nearby hotel accommodations were booked for others. These expenses and the risks of cross-infections between unvaccinated and immunocompromised patients, their families, and hospital and hotel staff became a challenge; particularly for patients coming from the Gaza Strip who required prolonged stays in hospital hotels (11). Finally, the political instability and violence against Palestinian health workers remain a threat to the response (2).

## Discussion

The high-risk setting in Palestine characterized by the physical, political, and economic barriers, protracted humanitarian situation in both the West Bank and Gaza, strains on the health system, increased economic degradation, high unemployment and mobility restrictions challenged AVH's response to COVID-19. Nevertheless, AVH's proactive and innovative preparedness and response strategies prevented the spread of COVID-19 among immunocompromised patients and enabled the maintenance of daily, continuous, essential, and critical cancer care. The early preparedness measures implemented by AVH before the first case reached Palestine, enabled its rapid adaptability and agility compared to other hospitals which began responding after the spread of the outbreak. AVH's designation as primary referral hospital by the Palestinian Authority, its strong leadership and coordination among the hospital networks and national authorities, and the representation of AVH's senior IPC specialist in the Minister of Health Committees on Epidemiology and COVID-19 response enabled knowledge-sharing, practical advising and mutual learning, in strengthening hospitals' outbreak preparedness in Palestine.

AVH contained nosocomial COVID-19 infections and maintained essential services for immunocompromised patients, following the latest guidelines provided by WHO (9). AVH reduced its capacity to 30% in March 2020 and within months managed to increase this to 50% by July 2020 to ensure the continuity of essential services for radiotherapy and dialysis patients across Palestine. Despite disruptions due to the ongoing political unrest along with the constraints of the pandemic, AVH's agility and resilience enabled MOH to optimize its referral pathways as one of three designated COVID-19 facilities nationally and the only hospital providing cancer care. Moreover, AVH reported that due to the above-mentioned measures, in the first outbreak in April 2020, they managed to contain it within 14 days without any cross-transmission between the high-risk dialysis and hematology departments. AVH further reported almost negligible incidence rates for IPC indicators, including 0 cases of ventilator-associated pneumonia (VAP) per 100 device days, a range of 0.0–0.3 catheter-associated urinary tract infection (CAUTI) cases per 1,000 device days, and a range of 0.0-7.4 central line-associated bloodstream infections (CLABSI) per 1,000 device days since Jan 2020-July 2021. In evaluating its ongoing response to COVID-19, AVH offers the following lessons:

1. **Proactive planning** was key; AVH was prepared to manage a COVID-19 outbreak before the first case was detected in the country. This allowed AVH to create and disseminate protocols, conduct drills, extensively train staff, and scale-up in-house surveillance capacities.
2. **Supportive leadership and multidisciplinary team approach in decision-making and clear coordination** allowed for swift actions and immediate implementation of preparedness and response strategies.
3. **Establishment of COVID-19 committee** evaluating epidemiological data, strategies, risks, and daily updates allowed the senior management team to make more **evidence-based decisions** and protect immunocompromised patients. **Facility-level surveillance** also strengthened the response.
4. **Daily provision of every health worker with personal protective equipment**, **along with training of health workers on their use**, **early and adequate procurement, and implementation of strict IPC measures, reduced the risk of nosocomial infection**. Changing AVH health worker attitudes and practices toward using PPE, even in social interactions, broke the cycle of transmission and prevented outbreaks in the hospital, despite national trends (10). This commitment to optimizing IPC especially in this high-risk setting was central to AVH's continued response and hospital operations (13).
5. **Continuous and clear communication** between hospital management and staff along with community engagement was central to creating, implementing, and evaluating the facility-level response plan.

These lessons learned are in line with regional findings, offering insights to the challenges, interventions, and lessons across each of the 10 hospital readiness domains, particularly from the EMR's low-and-middle income countries and countries in emergencies (16).

## Conclusion

AVH strengthened readiness and response by strengthening leadership and coordination, proactive and innovative logistical and supply chain management, flexible financing, horizontal and timely communication, health workforce safety and capacity building, rapid identification through facility-based screenings and agile triage, evidence-based clinical management, and prioritizing IPC (9, 13, 16). Despite the compounding challenges of instability, overcrowding, poverty, food insecurity, and disrupted procurement of medicines and supplies, AVH and Palestine's efforts to improve preparedness, reduce the impacts of the outbreak, ensure continuity of care for vulnerable populations, and vaccinate its citizens, offer invaluable lessons for other health systems in emergencies (10, 12, 14).

## Data Availability Statement

The original contributions presented in the study are included in the article/supplementary material, further inquiries can be directed to the corresponding author.

## Author Contributions

MK and HR: conception and drafting the article. MK, AS, MA, and HR: data collection, data analysis, and interpretation. MK, AS, MA, RP, AM, and HR: critical revision of the article and final approval of the version to be submitted.

## Conflict of Interest

The authors declare that the research was conducted in the absence of any commercial or financial relationships that could be construed as a potential conflict of interest.

## Publisher's Note

All claims expressed in this article are solely those of the authors and do not necessarily represent those of their affiliated organizations, or those of the publisher, the editors and the reviewers. Any product that may be evaluated in this article, or claim that may be made by its manufacturer, is not guaranteed or endorsed by the publisher.
